# Genetic Predisposition, Fruit Intake and Incident Stroke: A Prospective Chinese Cohort Study

**DOI:** 10.3390/nu14235056

**Published:** 2022-11-28

**Authors:** Jun Wang, Jianxin Li, Fangchao Liu, Keyong Huang, Xueli Yang, Xiaoqing Liu, Jie Cao, Shufeng Chen, Chong Shen, Ling Yu, Fanghong Lu, Liancheng Zhao, Ying Li, Dongsheng Hu, Jianfeng Huang, Dongfeng Gu, Xiangfeng Lu

**Affiliations:** 1 Department of Epidemiology, Fuwai Hospital, National Center for Cardiovascular Diseases, Chinese Academy of Medical Sciences and Peking Union Medical College, Beijing 100037, China; 2 Key Laboratory of Cardiovascular Epidemiology, Chinese Academy of Medical Sciences, Beijing 100037, China; 3 National Institute of Environmental Health, Chinese Center for Disease Control and Prevention, Beijing 100021, China; 4 Tianjin Key Laboratory of Environment, Nutrition and Public Health, Department of Occupational and Environmental Health, School of Public Health, Tianjin Medical University, Tianjin 300070, China; 5 Division of Epidemiology, Guangdong Provincial People’s Hospital and Cardiovascular Institute, Guangzhou 510080, China; 6 Department of Epidemiology and Biostatistics, School of Public Health, Nanjing Medical University, Nanjing 211166, China; 7 Department of Cardiology, Fujian Provincial Hospital, Fuzhou 350014, China; 8 Cardio-Cerebrovascular Control and Research Center, Institute of Basic Medicine, Shandong Academy of Medical Sciences, Jinan 250062, China; 9 Department of Epidemiology and Health Statistics, College of Public Health, Zhengzhou University, Zhengzhou 450001, China; 10 Department of Biostatistics and Epidemiology, School of Public Health, Shenzhen University Health Science Center, Shenzhen 518071, China; 11 School of Medicine, Southern University of Science and Technology, Shenzhen 518055, China

**Keywords:** polygenic risk score, fruit, stroke

## Abstract

The aim of this study was to evaluate the association between fruit intake and stroke risk considering the genetic predisposition. We used data from 34,871 participants from the project of Prediction for Atherosclerotic Cardiovascular Disease Risk in China (China-PAR project) from 2007 to 2020. A polygenic risk score comprising 534 genetic variants associated with stroke and its related factors was constructed to categorize individuals into low, intermediate, and high genetic risk groups. The associations of genetic and fruit intake with incident stroke were assessed by the Cox proportional hazard regression. We documented 2586 incident strokes during a median follow-up of 11.2 years. Compared with fruit intake < 200 g/week, similar relative risk reductions in stroke with adherence to fruit intake > 100 g/day across the genetic risk categories were observed (28–32%), but the absolute risk reductions were relatively larger in the highest genetic risk group (*p* for trend = 0.03). In comparison to those with a fruit intake < 200 g/week, those with a fruit intake >100 g/day in the low, intermediate, and high genetic risk groups had an average of 1.45 (95% CI, 0.61–2.31), 2.12 (1.63–2.59), and 2.19 (1.13–3.22) additional stroke-free years at aged 35, respectively. Our findings suggest that individuals with a high genetic risk could gain more absolute risk reductions and stroke-free years than those with a low genetic risk from increasing fruit intake for the stroke primary prevention.

## 1. Introduction

Stroke, caused by a combination of both a genetic predisposition and environmental risk factors, is the second-leading cause of death globally [1]. Notably, the stroke incidence is increasing over time in China, with a severe disease burden [2]. Fruit, including micronutrients and bioactive compounds, have been widely recommended in stroke prevention [3,4]. However, according to the China National Nutrition Surveys, only 4% of Chinese residents met the guideline-recommended level of fruit intake in 2012 [5]. Although there was a slight increase in average daily fruit intake among Chinese adults from 1982 to 2012, a low fruit intake still ranked as the second most common cause of stroke death [6].

Recent genome-wide association studies have identified a variety of genetic variants associated with stroke and its correlated traits, such as blood pressure (BP), type 2 diabetes, blood lipids, body mass index (BMI), etc. [7,8,9,10,11]. Subsequently, the polygenic risk score (PRS) was developed by combing multiple risk alleles to predict the risk of incident stroke [12,13,14]. Using PRS, some researchers estimated whether fruit intake could modify the effect of the genetic predisposition to stroke and its risk factors. Previous research in the USA found that increasing the fruit intake could attenuate the genetic predisposition on long-term weight gain [15]. However, the UK Biobank study failed to find an interaction between a genetic risk and a healthy diet, involving fruit intake, on stroke [12]. Further, it is still unclear whether the interaction on stroke existed among Chinese adults, and the extent to which an increased genetic predisposition to stroke can be offset by increasing the fruit intake, considering the different genetic backgrounds and dietary patterns from western populations.

Therefore, the aim of this study was to examine whether associations of fruit intake with the risk of stroke varied in different genetic risk groups among Chinese adults from the project of Prediction for Atherosclerotic Cardiovascular Disease Risk in China (China-PAR project).

## 2. Materials and Methods

### 2.1. Study Design and Participants

The study design and methods of the China-PAR project have been reported in detail, previously [16,17]. The study included three prospective cohorts with dietary records that were part of the China-PAR project, comprising the China Multi-Center Collaborative Study of Cardiovascular Epidemiology (China MUCA-1998), the International Collaborative Study of Cardiovascular Disease in Asia (InterASIA), and the Community Intervention of Metabolic Syndrome in China and Chinese Family Health Study (CIMIC). The baseline examination was 1998 for China MUCA-1998, 2000–2001 for InterASIA, and 2007–2008 for CIMIC, respectively. A follow-up survey was conducted for both the China MUCA-1998 and InterASIA cohorts during 2007 to 2008. Then all three cohorts were followed up in 2012–2015 and 2018–2020. The methods for the follow-up surveys were identical for all cohorts. The data collection was carried out after written informed consent was obtained from the participants, including an interviewer-administered questionnaire, physical examination, and blood sampling. This study was approved by the Institutional Review Board at Fuwai Hospital (Beijing, China). To avoid the influences of the economic development on fruit intake across the three cohorts enrolled at different periods [18], the survey in 2007–2008 with a unified questionnaire on dietary intake was used as the baseline in the present study. 

Among a total of 41,006 participants from three cohorts with available genotypic data [19], we further excluded 4856 individuals with missing information on fruit intake, 1265 with prevalent cardiovascular disease or cancer at 2007–2008, and 14 with missing data on follow-up, leaving 34,871 eligible participants in the final analysis.

### 2.2. Fruit Intake and Covariate Assessment

Dietary intake, including fruit intake, was collected by a simple standardized food-frequency questionnaire (FFQ), indicating acceptable reproducibility and validity in previous study [20]. Consumption frequency (daily, weekly, monthly, yearly, or never) and average consumption amount per unit of time for each food group during the previous year were reported by the participants, which were further converted into average daily intake. Processed fruit was excluded. Fruit intake was classified into three categories, based on the tertiles (<200 g/week, 200 g/week–100 g/day, and >100 g/day). The dietary score was computed, according to the consumption of four commonly eaten food groups in cardiometabolic health, based on current dietary guideline recommendations and our previous study evidence, including red meat, legumes, fish, and tea. The ideal levels were red meat < 75 g/day, legumes ≥ 125 g/day, fish ≥ 200 g/week, and tea ≥ 3 times/week [21,22]. The participants were given one point for each dietary component that reached the ideal level, otherwise they were given zero points. Based on this, we assigned equal weight for each food type and summed all points together, ranging from 0 to 4, with a higher index indicating a healthier dietary lifestyle.

The following variables were self-reported via standardized questionnaires, such as age, sex, region, urbanization, educational level, current smoking status, alcohol drinking status, physical activity, and medical conditions (use of antihypertensive, antidiabetic and lipid-lowering medications). Anthropometric measurements (height, weight, blood pressure, fasting blood glucose, and serum lipid levels) were performed following standard techniques by trained staff. Current smoking status was classified as smoker or nonsmoker by asking the participant whether he or she had smoked more than 100 cigarettes and whether he or she kept smoking up till then. Alcohol drinking was defined as alcohol consumption at least 12 times in the previous year. The physical activity level was evaluated by summing the time spent on moderate physical activity and on vigorous physical activity weighted by 2, and the ideal physical activity level was defined as at least 150 min/week of moderate physical activity or 75 min/week of vigorous physical activity or an equivalent combination. Body height and weight were measured twice by standardized anthropometric procedures with the participant wearing light garments and no shoes. The BMI was calculated as weight (kg)/height (m)^2^. Hypertension was diagnosed as mean systolic BP ≥ 140 mmHg and/or diastolic BP ≥ 90 mmHg (averaging three times standardized measurements with 30 s intervals in a seated position), and/or antihypertensive treatment in last two weeks. Overnight fasting blood samples were drawn for the measurements of serum glucose and lipid concentrations. Diabetes mellitus was defined as a fasting glucose level ≥ 126 mg/dL, and/or previous diagnosed diabetes, and/or antidiabetic treatment within two weeks. Subjects with a total cholesterol ≥ 240 mg/dL, or triglycerides ≥ 200 mg/dL, or low-density lipoprotein cholesterol ≥ 160 mg/dL, or high-density lipoprotein cholesterol < 40 mg/dL, or taking lipid-lowing medicine within two weeks were classified as dyslipidemia. 

### 2.3. Ascertainment of Incident Stroke Events

Information on stroke incidence was collected by well-trained staff to obtain medical records or death certificates from participants or their proxies. Local investigators initially recorded fatal and nonfatal stroke events. All medical and death records were reviewed by the central adjudication committee at Fuwai Hospital (Beijing, China) to determine the final diagnosis. Two adjudication committee members, blinded to the baseline information of the participants, verified the events independently, and any discrepancies were resolved by discussion with involvement of an additional committee member. Incident stroke was defined as a confirmed first ever fatal or nonfatal stroke event during the follow-up period, consisting of ischemic stroke (International Classification of Diseases, Tenth Revision I63), hemorrhagic stroke (I60–I62), and unspecified stroke (I64–I69) [19].

### 2.4. Polygenetic Risk Score for Stroke

The detailed information on single nucleotide polymorphisms (SNPs) selection and genotyping process used in the China-PAR study has been described, previously [19]. Briefly, a combined PRS was derived, based on 534 SNPs by using a meta-analytic approach and large genome-wide association results for stroke and stroke-related traits, including BP, type 2 diabetes, lipids, obesity, atrial fibrillation, and coronary artery disease, in East Asians. The detailed information on the selected SNPs is provided in Table S1. Genotyping for all participants was performed by multiplex PCR targeted amplicon sequencing technology. We designed multiplexed primers targeting each SNP and amplified the target regions for a high-throughput sequencing on a Hiseq X10 sequencer (Illumina, San Diego, CA, USA). Individual SNPs were coded as 0, 1, and 2 according to the number of risk alleles. The weight coefficient for each SNP was calculated in a previous publication. The PRS was formulated as the sum of the number of risk alleles at each locus multiplied by the respective weight coefficient, which was developed in a training set and validated in a validation set. The participants were categorized into low (lowest quintile of PRS), intermediate (quintiles 2 to 4 of PRS), and high (highest quintile of PRS) genetic risk groups, as described, previously [19].

### 2.5. Statistical Analysis

The baseline characteristics of the participants were described as mean (standard deviation) for continuous variables and numbers (percentages) for categorical variables. Age- and sex-adjusted incidence rates of stroke were calculated using the Poisson regression [23].

Person-years of follow-up were calculated from the date of the baseline examination to the date of the incident stroke, death, or follow-up interview for each study participant, whichever occurred first. To address the potential effect variation among the sub-cohorts, cohort-stratified Cox proportional hazards models with the duration of the follow-up as the time metric were applied to estimate the hazard ratios (HRs) and their 95% confidence intervals (CIs) of incident stroke, with the lowest category of genetic risk score or fruit intake as reference. Proportional hazards assumptions were not violated when assessed using the Schoenfeld residuals (*p* > 0.05). The selection process of the potential confounders was based on an extensive literature search and then tested by a univariate Cox regression analysis. Two models were constructed to account for the potential confounders. The base model included age and sex for the genetic risk association analysis, as previously described [19], and included age, sex, region, urbanization, and educational level, for the fruit intake association analysis. The full model further included the current smoking status, alcohol drinking status, physical activity, BMI, diet score, and vegetables intake. The tests of trend were conducted by modeling the median of the PRS or fruit intake of each category, as a continuous variable. Potential nonlinear relationships of incident stroke, associated with the PRS and fruit intake were assessed by restricted cubic splines with three knots at the 25th, 50th (reference), and 75th percentiles of the distribution.

Moreover, we investigated the combined effects of the PRS and fruit intake on stroke, with the lowest genetic risk and fruit intake > 100 g/day, as reference. We further explored their potential interaction between the PRS and fruit intake. To test for the multiplicative interaction, we included a multiplicative interaction term in the full model. An additive interaction was also tested by calculating the relative excess risk due to the interaction (RERI) and the attributable proportion due to the interaction (AP) [24].

We calculated the cumulative incidence of stroke for three categories of fruit intake within each genetic risk stratum. The absolute risk reductions (ARRs) in 10-year stroke incidence between low and high fruit intake groups were computed, and their trends across the genetic risk categories were tested using the weighted least-squares model [19].

The gained stroke-free years related to fruit intake were estimated as the differences of the areas under the survival curves, based on the fully-adjusted Cox proportional hazard model with age as the underlying timescale, and 95% CIs were derived by drawing 500 bootstrap samples from the estimation dataset [25].

To examine the robustness of our results, we performed several sensitivity analyses by (1) using the chronological age as the primary timescale instead of the time-on-study; (2) accounting for competing the risk of a non-stroke death using Fine and Gray’s approach [26]; (3) adjusting for the smoking packs per year and daily alcohol intake as continuous variables instead of categorical variables; (4) further adjusting for the family history of stroke or per-capita household income; (5) separately investigating the interaction between each SNP in the PRS and fruit intake.

We additionally estimated the association of the frequent intake of fruit with stroke risk stratified by the genetic risk category. All statistical analyses were performed using SAS software, version 9.4 (SAS Institute, Cary, NC, USA) and R software, version 4.2.1 (R Foundation for Statistical Computing, Vienna, Austria). The R package “forestplot”, “cmprsk”, and “survival” were used. A two-sided *p* value < 0.05 indicated statistical significance.

## 3. Results

During a median of 11.2 years of follow-up, we documented 2586 first-ever incident stroke events, including 1834 ischemic stroke, 439 hemorrhagic stroke, 55 both ischemic and hemorrhagic stroke, and 258 strokes with an unknown subtype, with the incidence of 7.7 per 1000 person-years. The baseline characteristics of the study population, stratified according to the presence of incident stroke are shown in Table 1, and the characteristics according to the genetic risk category and fruit intake are presented in Table S2. The mean age of the participants eligible for the analysis was 55 ± 10 years, and 58% were female. The average fruit intake was 83 g/day. Compared to the non-cases, the stroke cases were more likely to be older, had a higher BMI and waist circumference, a higher baseline prevalence of hypertension, diabetes mellitus and dyslipidemia, a poorer ideal level of diet score, and a lower fruit and vegetable intake.

The univariate Cox regression analysis results of potential confounders were shown in Table S3. The genetic risk score showed a normal distribution (Figure S1). The stroke risk increased monotonically across the range of the genetic risk score (*p* overall < 0.001, *p* nonlinear = 0.37). Compared with those with a low genetic risk score, the age- and sex-adjusted HRs (95% CIs) for the intermediate and high genetic risk groups were 1.19 (1.06–1.32) and 1.51 (1.33–1.71) when only adjusted for age and sex, respectively (Table S4). The results were basically unchanged by the adjustment for other factors. Conversely, a higher fruit intake was associated with a lower risk of stroke (*p* overall < 0.001, *p* nonlinear = 0.02). Based on the fully adjusted model, the participants with a fruit intake of 200 g/week–100 g/day and >100 g/day had 19% (HR, 0.81; 95% CI, 0.74–0.89) and 31% (HR, 0.69; 95% CI, 0.62–0.77) lower risk of stroke, respectively, compared with those consuming less than 200 g/week.

**Figure 1:**
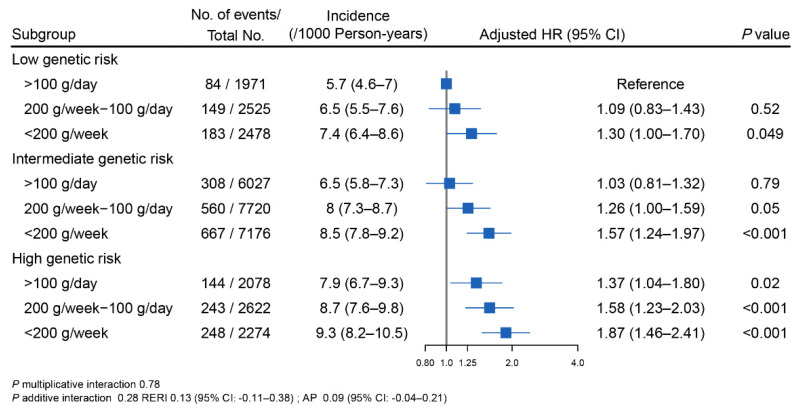
The joint association of genetic risk and fruit intake with incident stroke. Age- and sex-adjusted incidence rates per 1000 person-years of stroke were calculated using the Poisson regression. The overall genetic risk for stroke was defined as high (quintile 5 of PRS), intermediate (quintile 2–4 of PRS), and low (quintile 1 of PRS). In these comparisons, the participants with a low genetic risk and a fruit intake > 100 g/day served as the reference group. HRs were derived from the Cox proportional hazards models stratified by cohort and adjusted for age (continuous), sex (male, female), region (North/South China), urbanization (urban, rural), education level (less than high school, high school, or above), current smoking status (yes, no), alcohol drinking status (yes, no), physical activity (continuous), body mass index (continuous), diet score (continuous), and vegetables intake (daily intake ≥ 500 g/day or not). Multiplicative interaction was evaluated using the hazard ratios for the product term between the fruit intake (<200 g/week vs. >100 g/day) and the PRS (low vs. high). *p* multiplicative interaction was 0.78. To estimate the additive interaction, the participants with a fruit intake of ≥200 g/week and the low genetic risk (quintile 1 of PRS) were used as the reference with consideration of the practical interpretations. Two indexes were calculated: the relative excess risk due to the interaction (RERI) and the attributable proportion due to the interaction (AP). *p* additive interaction was 0.28. RERI was 0.13 (95% CI: −0.11–0.38). AP was 0.09 (95% CI: −0.04–0.21). Abbreviations: CI, confidence interval; HR, hazard ratio; PRS, polygenic risk score.

When the genetic risk and fruit intake were combined, the overall risk of incident stroke increased as both the genetic risk and unfavorable fruit intake increased (Figure 1). Specifically, the age- and sex-adjusted incidence rates of stroke per 1000 person-years ranged from 5.7 (95% CI, 4.6–7) for participants with a low genetic risk and a high fruit intake to 9.3 (95% CI, 8.2–10.5) for participants with a high genetic risk and a low fruit intake. Participants with a high genetic risk and a low fruit intake had the highest risk of incident stroke, with a HR of 1.87 (95% CI, 1.46–2.41). No significant multiplicative or additive interactions between the PRS and fruit intake on stroke were recorded.

**Figure 2:**
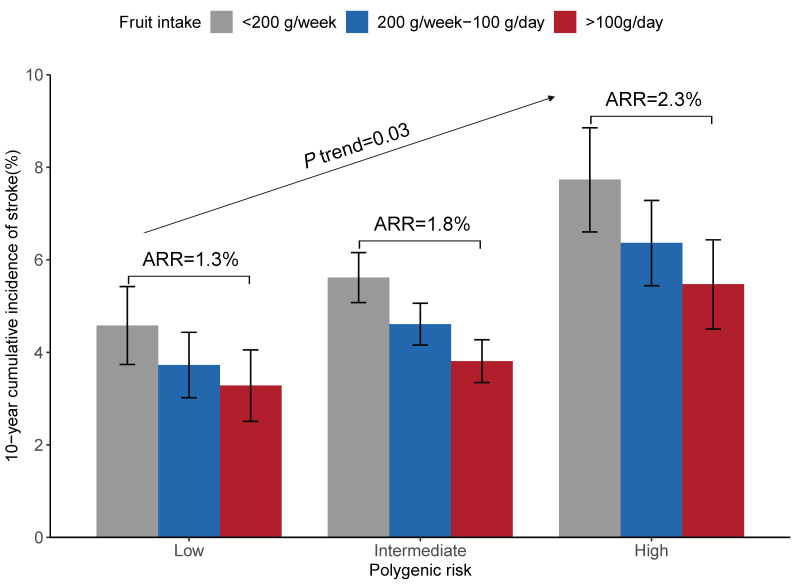
Adjusted 10-year cumulative incidence of stroke according to the genetic risk and fruit intake. Standardized to the means of cohort, age, sex, region, urbanization, education level, current smoking status, alcohol drinking status, physical activity, body mass index, diet score, and vegetables intake within the study population. Abbreviations: ARR, absolute risk reduction.

Further analyses stratified by the genetic risk category with a fruit intake less than 200 g/week as the reference group confirmed that a higher fruit intake was associated with a lower risk of stroke within each category of genetic risk, with adjusted HRs (95% CIs) of 0.72 (0.54–0.95), 0.68 (0.59–0.78), and 0.70 (0.56–0.87) among the participants in the low, intermediate, and high genetic risk groups, respectively (Figure S2). However, in terms of the absolute benefit of increasing the fruit intake, there was a significant gradient of ARRs across the low, intermediate, and high genetic risk categories (1.3%, 1.8%, and 2.3%, *p* for trend = 0.03, Figure 2).

**Figure 3:**
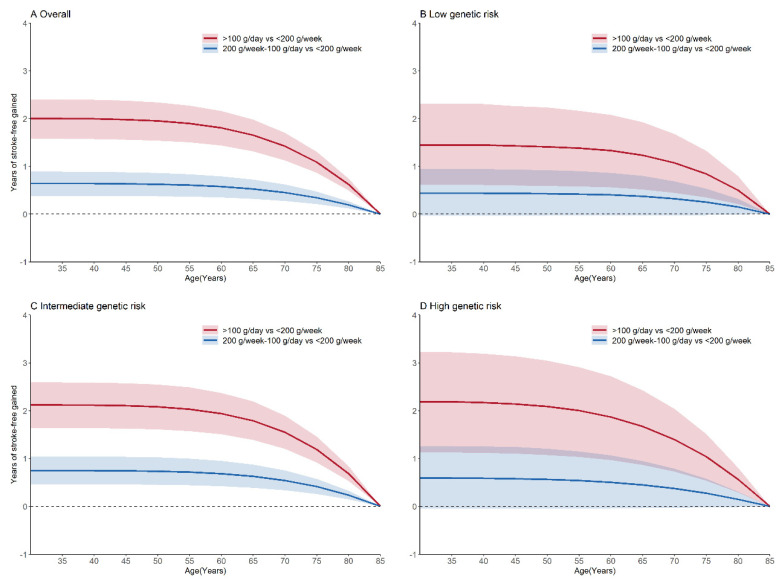
Stroke-free years gained associated with the fruit intake. The overall genetic risk for stroke was defined as high (quintile 5 of PRS), intermediate (quintile 2–4 of PRS), and low (quintile 1 of PRS). Models were adjusted for the cohort, age, sex, region, urbanization, education level, current smoking status, alcohol drinking status, physical activity, body mass index, diet score, and vegetables intake. Abbreviations: PRS, polygenic risk score.

We also estimated the gains of the stroke-free years of increment in the fruit intake under the overall and the three genetic risk groups. In participants aged 35 years, a fruit intake of 200 g/week–100 g/day and >100 g/day were associated with an average of 0.64 (95% CI, 0.38–0.89) and 2 (95% CI, 1.57–2.4) additional stroke-free years, respectively, compared to a fruit intake of less than 200 g/week (Figure 3). The additional stroke-free years of a fruit intake >100 g/day were 1.45 (95% CI, 0.61–2.31), 2.12 (95% CI, 1.63–2.59), and 2.19 (95% CI, 1.13–3.22) for the participants in the low, intermediate, and high genetic risk groups, respectively, as compared with a fruit intake of less than 200 g/week at the age of 35.

The results were consistent in a series of sensitivity analyses (Table S5). For the interactions between the individual SNPs and fruit intake, two SNPs (rs12999907 at *AC092684.1* and rs2531995 at *ADCY9*) showed significant additive interactions with *p* < 0.001, however, they would not reach significance after a Bonferroni adjustment (*p* < 0.05/534) (Table S1). Moreover, a significant gradient of the absolute benefit from the daily consumption of fruit across the low, intermediate, and high polygenic risk categories was also observed (*p* trend = 0.03, Table S6).

## 4. Discussion

Using a large-scale Chinese prospective cohort study, an increased PRS was positively associated with a stroke risk. Adherence to a higher fruit intake was associated with a reduced risk of developing stroke and the absolute risk reduction was most evident among those at the highest genetic risk. It was also estimated that increasing the fruit intake from less than 200 g/week to above 100 g/day resulted in longer stroke-free years among those with a high genetic risk, compared with those with a low genetic risk at the age of 35 years.

Our findings of the positive influence of adherence to an increased fruit intake to prevent stroke are generally comparable with the previous studies [3,4,27,28,29]. However, the findings from the Prospective Urban Rural Epidemiology (PURE) did not find an association between fruit intake and incident stroke [30]. The difference in the types of fruit consumed may partly explain the negative result in the PURE study, which were also found in an early study [31]. Moreover, the association between the fruit intake and stroke risk in individuals with different genetic burdens for stroke were not evaluated in these studies. Although two prior studies showed that the increased cardiovascular disease risk associated with the genetic variants in the chromosome 9p21 region, such as rs2383206 and rs4977574, appeared to be modified by the adherence to a higher fruit and vegetable intake [32,33], population-based studies that qualify the potential interactions of the aggregated genetic susceptibility and fruit intake with stroke risk were rather limited.

With the advances in the field of nutritional genetics, some researchers cast doubt on the “one size fits all” public Dietary Reference Intakes for individuals. The reality of nutrition and dietetic practice is complicated. Accordingly, it is time to unravel the degree to which the diet influences the disease development, and whether it may depend more on a person’s genetic background [34,35]. The UK Biobank study reported that a healthy diet including a fruit intake could not decrease the stroke risk across all genetic risk categories, using 11 SNPs [36]. However, a significant relative risk of incident stroke associated with an unhealthy diet was merely observed among the high genetic risk group when extending to 90 SNPs [12]. That might be explained by the high predictive power due to the inclusion of more SNPs. Consistent with our study, no evidence was identified for the interaction between the genetic risk and adherence to a healthy diet, including the fruit intake [12]. Nonetheless, it is worth noting that individuals with the highest burden of genetic risk derived the greatest absolute risk reductions and largest gain in stroke-free years with a fruit intake >100 g/day, in the present study, which may provide evidence for the beneficial effects of increasing the fruit intake on stroke prevention among Chinese populations, especially for those with a high genetic risk.

The potential mechanisms that an increased fruit intake might be beneficial to cardiovascular health have been suggested. Fruit provides excellent sources of fiber, potassium, micronutrients, and bioactive compounds which are necessary for human health. The involved biological mechanisms included protecting the vascular endothelial function, regulating lipids metabolism, modulating blood pressure, alleviating ischemia/reperfusion injury, suppressing thrombosis, reducing oxidative stress, and attenuating inflammation [37].

The strengths of this study included its large sample size, long-term follow-up period, and the strict and comprehensive data collection. We first estimated the interaction of the genetic risk and fruit intake on incident stroke, as well as stroke-free years by increasing the fruit intake among the Chinese population. Our results were robust to a variety of sensitivity analyses. Nevertheless, the present study has several limitations. First, the fruit intake was assessed by a simple FFQ. A recall bias of self-reported dietary information is of concern, which might lead to misclassification. However, the FFQ has been well validated among the Chinese population [20]. Second, the total fruit were analyzed with a lack of the information on different types of fruit available in the present study. Hence, it is still not clear which types of fruit may be the most relevant in gene-diet interactions, which need further studies. Third, participants with increased fruit consumption are likely to have a relatively healthier lifestyle. Although we adjusted for several major lifestyle and dietary factors in the analysis, some potential confounders, such as salt or sugar intake (not measured in this study), may modulate the genetic association with stroke. Fourth, a change in fruit intake over the follow-up period was not evaluated, and future studies would be warranted. Fifth, due to the limited number of hemorrhagic stroke cases and the heterogeneity in the influence of the genetic variants on different stroke subtypes, the associations of fruit intake and subtype-specific stroke genetic risk scores are not evaluated [14]. Finally, our study was restricted to the Chinese population, and further study is needed to evaluate the generalizability of our stroke PRS to other East Asian populations.

## 5. Conclusions

In summary, increasing the fruit intake was associated with a significantly decreased risk of stroke, and individuals with a high genetic risk derived greater benefit from increasing their fruit intake. Our study supports the recommendations of the adherence to fruit intake for the reduction of strokes in the Chinese population, particularly in those at a high genetic risk.

## Figures and Tables

**Table 1:** Baseline characteristics of the participants.

Characteristic	No Incident Stroke	Incident Stroke	*p* Value
	(*n* = 32,285)	(*n* = 2586)	
Age, year	55 ± 10	60 ± 9	<0.001
Female, *n* (%)	18,701 (58)	1427 (55)	0.007
Northern, *n* (%)	15,389 (48)	1437 (56)	<0.001
Urban residence, *n* (%)	5462 (17)	151 (5.8)	<0.001
High-school or above, *n* (%)	15,544 (48)	779 (30)	<0.001
Current smoker, *n* (%)	7560 (23)	633 (25)	0.22
Alcohol drinker, *n* (%)	7571 (23)	521 (20)	<0.001
Family history of stroke, *n* (%)	3188 (10)	303 (12)	0.003
Ideal physical activity, *n* (%)	20,244 (63)	1662 (64)	0.11
Ideal diet score, *n* (%)	17,019 (53)	1086 (42)	<0.001
Body mass index, kg/m^2^	24 ± 4	24 ± 4	<0.001
Waist circumference, cm	82 ± 10	84 ± 10	<0.001
Hypertension, *n* (%)	11,945 (37)	1435 (56)	<0.001
Diabetes mellitus, *n* (%)	2596 (8.3)	329 (13)	<0.001
Dyslipidemia, *n* (%)	10,047 (32)	1025 (41)	<0.001
Fruit intake, g/day	85 ± 86	66 ± 78	<0.001
Vegetable intake, g/day	335 ± 157	327 ± 158	0.02

Data are mean ± standard deviation for the continuous variables and numbers (percentages) for the dichotomous variables. Ideal physical activity level was defined as at least 150 min/week of moderate physical activity or 75 min/week of vigorous physical activity or an equivalent combination. The ideal diet was defined as a healthy diet score ≥ 2 components: red meat < 75 g/day, legumes ≥ 125 g/day, fish ≥ 200 g/week, and tea ≥ 3 times/week.

## Data Availability

All data supporting the findings of this study are available within the article and its supplementary information files. The datasets used and analyzed during the current study are available from the corresponding author upon reasonable request.

## Supplementary Materials

The following supporting information can be downloaded at: https://www.mdpi.com/article/10.3390/nu14235056/s1, Table S1. Information about the selected SNPs and the interplay between a single SNP and fruit intake. Table S2. Baseline characteristics by the genetic risk score and the daily fruit intake. Table S3. Association of the stroke risk with the sociodemographic and behavioral factors. Table S4. Multivariable-adjusted HRs (95% CIs) for genetic risk, fruit intake, and incident stroke. Table S5. Sensitivity analysis of the fruit intake associated with incident stroke, according to the genetic risk category. Table S6. Risk of incident stroke, according to the fruit intake frequency and genetic risk. Figure S1. Associations between the genetic risk score and incident stroke. Figure S2. Cumulative incidence of stroke, according to the categories of the genetic risk and fruit intake.
