# Supplementary material for: Genetic Predisposition, Fruit Intake and Incident Stroke: A Prospective Chinese Cohort Study
Source: Nutrients. 2022 Nov 28;14(23):5056. doi: 10.3390/nu14235056 (PMC9740837; doi:10.3390/nu14235056)
Supplement: Supplementary file 1 [file nutrients-14-05056-s001.zip › nutrients-2011062-supplementary.pdf]

Supplementary Material

Additional details of fruit intake assessment

The trained and certificated interviewer asked each participant to report how often, on average, they have consumed each food item on a daily, weekly, monthly, yearly, or never scale over the past 12 months at baseline and during follow-up visits, followed by a question on the amount consumed in liang (1 liang=50 g) per unit of time. The food items include fresh fruit, fresh vegetables, legumes, egg, fish, red meat, milk, poultry meat, salted vegetables, nut, and tea. Combined with intake amounts, the intake frequencies were converted into the average daily intake for each food item. Processed fruit was excluded.

Additional details of SNP selection and PRS construction

We selected 588 single-nucleotide polymorphisms (SNPs) that showed genome-wide significant associations ($P < 5\times10^{-8}$) with stroke and its-related traits, including blood pressure (BP), coronary artery disease (CAD), type 2 diabetes (T2D), lipids, obesity, and atrial fibrillation (AF), based on previous genome-wide association study (GWASs).

The selected SNPs associated with stroke and CAD were reported amongst East Asian and European populations due to the limited number of loci identified in East Asian individuals. For other traits, we mainly included the reported SNPs in East Asian ancestry. Genotyping for all participants was performed by multiplex PCR targeted amplicon sequencing technology. We designed multiplexed primers targeting each SNP and amplified the target regions for high-throughput sequencing on a Hiseq

X10 sequencer (Illumina). Finally, a total of 534 SNPs remained for the present analysis after strict quality control.

The number of SNPs included

Traits	Number of SNPs
Stroke	42
Blood pressure (systolic BP, SBP; diastolic BP, DBP; purse pressure, PP; mean arterial pressure, MAP)	46
Coronary artery disease	199
Type 2 diabetes	89
Obesity (body mass index, BMI; waist circumference, WC; waist hip ratio, WHR)	79
Lipids (total cholesterol, TC; triglyceride, TG; low density lipoprotein cholesterol, LDL-C; high density lipoprotein cholesterol, HDL-C)	126
Atrial fibrillation	16
Total	588

Fourteen trait-specific PRSs (stroke, CAD, T2D, AF, SBP, DBP, PP, MAP, BMI, WC, TC, TG, LDL-C, and HDL-C) were calculated separately by adding the number of corresponding risk alleles (0,1,2) weighted by the effect size of each SNP on each trait. For stroke, the effect sizes were extracted from summary statistics of the largest stroke GWAS conducted by Biobank Japan (17,671 stroke cases and 192,383 controls). For stroke-related traits, the effect size data were drawn from large-scale GWASs for each trait conducted among East Asian individuals, and those containing Chinese samples were selected in priority if more than one GWAS were reported. Details of utilized GWAS studies were published elsewhere.

For each trait, we constructed 16 candidate PRS using different threshold values for linkage disequilibrium (LD) metric r^2 (0.2, 0.4, 0.6, 0.8) and P value (0.5, 0.05, 5×10^{-4}, 5×10^{-6}) based on trait-specific summary statistics from the large-scale GWAS.

The score with the largest magnitude of odds ratio (OR) (per SD increase of PRS) for stroke in the training set (case control study design in China, 2872 stroke cases and 2494 controls) was selected as the optimal trait-specific PRS. Each optimal PRS was standardized to zero mean and unit SD. The elastic-net logistic regression with 10-fold cross-validation was used to model the associations between the 14 optimal PRS and stroke and further to generate the metaPRS.

Table S1. Information about the selected SNPs and the interplay between single SNP and fruit intake

SNP	Chr: Pos_hg19	Gene	Effect allele	MAF in cohort	Trait	*P* multiplicative interaction	*P* additive interaction
rs880315	1:10796866	CASZ1	T	0.38	BP;stroke	0.15	0.1
rs7528419	1:109817192	CELSR2	G	0.06	CAD	0.83	0.49
rs12740374	1:109817590	CELSR2	T	0.06	LDL-C;TC	0.7	0.37
rs12037987	1:113042822	WNT2B	C	0.3	stroke	0.34	0.21
rs10745332	1:113189053	CAPZA1	G	0.18	BP	0.3	0.59
rs17030613	1:113190807	CAPZA1	C	0.47	BP	0.21	0.51
rs4846049	1:11850365	MTHFR	T	0.17	BP	0.2	0.48
rs984222	1:119503843	TBX15	C	0.4	WHR	0.21	0.01
rs10923931	1:120517959	NOTCH2	T	0.03	T2D	0.78	0.15
rs11810571	1:151762308	TDRKH	G	0.5	CAD	0.8	0.58
rs67156297	1:154336716	RP11-350G8.4	A	0.09	T2D	0.74	0.9
rs4845625	1:154422067	IL6R	T	0.49	CAD	0.39	0.1
rs6666258	1:154814268	KCNN3	C	0.03	AF	0.51	0.74
rs1052053	1:156202173	PMF1-SEMA4A	G	0.34	stroke	0.38	0.88
rs2758607	1:156202759	PMF1-BGLAP	A	0.34	stroke	0.45	0.9
rs1892094	1:169094459	ATP1B1	T	0.06	CAD	0.72	0.98
rs1200159	1:169100241	ATP1B1	T	0.17	CAD	0.1	0.62
rs3903239	1:170569317	RP1-79C4.1	A	0.4	AF	0.12	0.8
rs2213732	1:172333595	DNM3	G	0.13	WHR	0.94	0.14
rs574367	1:177873210	SEC16B	T	0.2	BMI	0.25	0.66
rs1689800	1:182168885	LINC01344	G	0.3	HDL-C	0.69	0.76
rs6700559	1:200646073	RP11-92G12.3	C	0.31	CAD	0.12	0.04
rs2820315	1:201872264	LMOD1	T	0.12	CAD	0.52	0.48
rs2819348	1:201884952	LMOD1	C	0.12	CAD	0.86	0.6
rs60154123	1:210468999	RP4-667H12.4	T	0.29	CAD	0.62	0.44
rs2075423	1:214154719	PROX1	T	0.17	T2D	0.21	0.49
rs340874	1:214159256	PROX1	C	0.39	T2D	0.5	0.73
rs2820443	1:219753509	RP11-95P13.2	C	0.29	WHR	0.79	0.74
rs2642442	1:220973563		C	0.15	TC;LDL-C	0.45	0.79
rs17465637	1:222823529	MIA3	A	0.39	CAD	0.71	0.33
rs67180937	1:222823743	MIA3	T	0.42	CAD	0.6	0.45
rs36096196	1:2252205	MORN1	T	0.14	CAD	0.98	0.55
rs2144300	1:230294916	GALNT2	T	0.18	TG	0.87	0.53
rs699	1:230845794	AGT	A	0.19	CAD	0.5	0.9
rs2783963	1:243501583	SDCCAG8	A	0.08	BMI	0.4	0.64
rs12027135	1:25775733	TMEM57	T	0.31	TC;LDL-C	0.43	0.47
rs61776719	1:38461319	SF3A3	A	0.33	CAD	0.37	0.79
rs2296172	1:39835817	MACF1	G	0.17	HDL-C	0.42	0.39
rs11205760	1:51174330	FAF1	C	0.22	T2D	0.21	0.18

rs11206510	1:55496039	PCSK9	C	0.06	CAD	0.45	0.62
rs7525649	1:55499156	PCSK9	C	0.35	LDL-C;TC	0.12	0.36
rs151193009	1:55509585	PCSK9	T	0.02	LDL-C;TC	0.96	0.99
rs9970807	1:56965664	PLPP3	T	0.03	CAD	0.08	0.25
rs12042319	1:63049819	DOCK7	A	0.2	TC;TG	0.35	0.82
rs995000	1:63107526	DOCK7	T	0.19	TG	0.4	0.85
rs10889353	1:63118196	DOCK7	C	0.19	TC;TG	0.4	0.83
rs2815752	1:72812440	RPL31P12	G	0.08	BMI	0.48	0.88
rs1514175	1:74991644	FPGT-TNNI3K	G	0.23	BMI	0.28	0.25
rs17381664	1:78048331	ZZZ3	C	0.01	BMI	0.54	0.69
rs117711462	1:93159927	EVI5	A	0.01	LDL-C;TC	0.52	0.33
rs1555543	1:96944797	EEF1A1P11	A	0.13	BMI	0.52	0.76
rs17678683	2:145286559	ZEB2	G	0.05	CAD	0.61	0.61
rs7560163	2:151637936	AC104777.4	G	0.1	T2D	0.09	0.37
rs4377290	2:158437683	ACVR1C	C	0.31	TC	0.18	0.76
rs16849225	2:164906820	AC092684.1	T	0.4	BP	0.03	0.02
rs12999907	2:164957251	AC092684.1	G	0.37	CAD	0.04	<0.001
rs12692735	2:165504565	COBLL1	T	0.1	T2D	0.34	0.68
rs840616	2:188196469	AC007319.1	T	0.11	CAD	0.47	0.16
rs1344653	2:19730845	LINC01808	G	0.18	BP	0.78	0.93
rs16986953	2:19942473	AC019055.1	A	0.35	CAD	0.28	0.97
rs2123536	2:19945577	AC019055.1	T	0.37	CAD	0.08	0.92
rs6725887	2:203745885	WDR12	C	0.02	CAD	0.52	0.55
rs13306194	2:21252534	APOB	A	0.12	LDL-C;TC;TG	0.2	0.85
rs1367117	2:21263900	APOB	A	0.13	TC;LDL-C	0.17	0.1
rs515135	2:21286057	APOB	T	0.11	CAD	0.94	0.29
rs312949	2:21334283	TDRD15	C	0.27	LDL-C;TC	0.29	0.38
rs17517928	2:216291359	FN1	T	0	CAD	0.64	0.15
rs1250229	2:216304384	FN1	T	0.09	CAD	0.58	0.62
rs2571445	2:218683154	TNS1	A	0.4	CAD	0.98	0.26
rs2972146	2:227100698	NEU2	G	0.07	CAD	0.87	0.32
rs2972143	2:227116365	NEU2	A	0.07	HDL-C	0.38	0.21
rs11677932	2:238223955	STK25	A	0.35	CAD	0.4	0.76
rs6545814	2:25131316	ADCY3	G	0.42	BMI	0.59	0.99
rs736699	2:26913930	KCNK3	G	0.24	stroke	0.39	0.96
rs1275988	2:26914364	KCNK3	T	0.24	BP	0.28	0.89
rs1260326	2:27730940	GCKR	C	0.47	TC;TG	0.99	0.3
rs780094	2:27741237	GCKR	C	0.48	T2D	0.99	0.45
rs10203174	2:43690030	THADA	T	0.01	T2D	0.29	0.08
rs35419456	2:43725965	THADA	A	0.04	T2D	0.83	0.38
rs6544713	2:44073881	ABCG8	T	0	CAD	0.34	0.21
rs582384	2:45896437	PRKCE	A	0.25	CAD	0.19	0.25
rs9309245	2:53397048	AC010967.2	G	0.19	T2D	0.16	0.02
rs3791679	2:56096892	EFEMP1	A	0.21	WC	0.09	0.2

rs1116357	2:57287411	RP11-443I9.1	G	0.24	T2D	0.04	0.04
rs1861411	2:58904177	LINC01122	A	0.41	BMI	0.18	0.7
rs243019	2:60585806	MIR4432HG	T	0.32	T2D	0.7	0.34
rs11125936	2:62871225	AC092155.4	C	0.22	LDL-C	0.25	0.7
rs12463617	2:629244	TMEM18	A	0.09	BMI	0.92	0.46
rs2861568	2:67571721	AC023115.4	T	0.49	WHR	0.14	0.55
rs7568458	2:85788175	GGCX	A	0.39	CAD	0.58	0.8
rs7616006	3:12267648	LINC00690	G	0.35	TC	0.1	0.98
rs79223353	3:123139863	ADCY5	A	0.37	T2D	0.76	0.32
rs1801282	3:12393125	PPARG	G	0.06	T2D	0.59	0.88
rs17843768	3:124448385	UMPS	A	0.08	CAD	0.73	0.31
rs17843797	3:124453022	UMPS	G	0.08	CAD	0.84	0.28
rs2625967	3:129267259	H1FOO	G	0.4	WHR	0.23	0.09
rs10512861	3:132257961		T	0.12	CAD	0.19	0.39
rs667920	3:136069472	STAG1	G	0.13	CAD	0.82	0.76
rs6807945	3:138052754	NME9	C	0.23	CAD	0.82	0.16
rs9818870	3:138122122	MRAS	T	0.01	CAD	0.44	0.95
rs7610618	3:149157706	SIAH2	T	0.04	stroke	0.54	0.17
rs748431	3:14928077	FGD5	T	0.31	CAD	0.27	0.75
rs1868673	3:150187314	TSC22D2	A	0.49	WC	0.79	0.76
rs4266144	3:156852592	SPTSSB	C	0.32	CAD	0.65	0.85
rs12897	3:172115902	FNDC3B	A	0.26	CAD	0.68	0.47
rs1470579	3:185529080	IGF2BP2	C	0.26	T2D	0.59	0.11
rs10513801	3:185822353	ETV5	G	0.04	BMI	0.12	0.11
rs17301514	3:186613409	RPS20P14	A	0.05	T2D	0.89	0.56
rs6808574	3:187740523	TMEM207	T	0.01	T2D	0.18	0.06
rs1496653	3:23454790	UBE2E2	G	0.21	T2D	0.13	0.39
rs820430	3:27548900	FECHP1	A	0.32	BP	0.09	0.13
rs9815354	3:41912651	ULK4	A	0.19	BP	0.37	0.78
rs7633770	3:46688562	SNORD77	A	0.23	CAD	0.46	0.68
rs7617773	3:48193515	TKT	C	0.31	CAD	0.65	0.61
rs2535633	3:52859630	ITIH4	G	0.41	BMI	0.35	0.9
rs9810888	3:53635595	CACNA1D	G	0.4	BP	0.36	0.003
rs9828933	3:64002897	PSMD6	C	0.31	T2D	0.5	0.57
rs6795735	3:64705365	ADAMTS9-AS2	C	0.25	T2D	0.65	0.89
rs13078807	3:85884150	CADM2	G	0	BMI	0.1	0.32
rs6825911	4:111381638	ENPEP	C	0.47	BP	0.22	0.48
rs1448818	4:111570223	RP11-119H12.3	C	0.36	AF	0.86	0.56
rs6817105	4:111705768	RP11-119H12.3	T	0.48	AF	0.52	0.93
rs2200733	4:111710169	RP11-119H12.3	C	0.49	stroke	0.48	0.82

rs13143308	4:111714419	LINC01438	G	0.28	stroke	0.82	0.82
rs4400058	4:111716673	LINC01438	A	0.2	AF	0.42	0.67
rs6838973	4:111765495	RP11-119H12.3	C	0.39	AF	0.63	0.43
rs13115759	4:113710461	RPL7AP30	A	0.35	stroke	0.36	0.71
rs10010670	4:113711748	RPL7AP30	G	0.35	stroke	0.37	0.8
rs7678555	4:120909501	RP11-170N16.1	C	0.21	CAD	0.52	0.79
rs73069940	4:1236502	CTBP1	G	0.34	T2D	0.21	0.88
rs4593108	4:148281001	FHDC1	G	0.33	CAD	0.47	0.75
rs17612742	4:148414651	EDNRA	C	0.22	stroke	0.12	0.06
rs6813195	4:153520475	FHDC1	T	0.45	T2D	0.08	0.85
rs6825454	4:155501188	FGA	C	0.47	stroke	0.24	0.61
rs1976041	4:156486040	TOMM22P4	A	0.23	CAD	0.57	0.66
rs13143871	4:156619204	GUCY1A3	C	0.21	BP	0.28	0.02
rs72689147	4:156639888	GUCY1A3	T	0.19	CAD	0.43	0.02
rs7696431	4:169687725	PALLD	T	0.45	CAD	0.74	0.51
rs6818397	4:3434885	RGS12	T	0.49	TG	0.14	0.81
rs16844401	4:3449652	HGFAC	A	0.09	CAD	0.72	0.34
rs6831256	4:3473139	DOK7	G	0.36	TG	0.16	0.75
rs16858082	4:45175804	NMU	T	0.34	BMI	0.43	0.35
rs871606	4:54799245	RP11-231C18.3	C	0.19	BP	0.83	0.76
rs17087335	4:57838583	NOA1	T	0.4	CAD	0.41	0.68
rs4458523	4:6289986	WFS1	T	0.05	T2D	0.99	0.53
rs12500824	4:77416627	SHROOM3	A	0.45	CAD	0.73	0.66
rs1902859	4:81157703	RP11-576N17.4	C	0.41	BP	0.38	0.2
rs10857147	4:81181072	RP11-576N17.4	T	0.4	CAD	0.04	0.12
rs11099493	4:82587050	RASGEF1B	G	0.2	CAD	0.61	0.36
rs6829822	4:86716496	ARHGAP24	T	0.18	BP	0.47	0.41
rs1037814	4:88049850	AFF1	T	0.42	TG	0.87	0.79
rs3775058	4:96117371	UNC5C	T	0.49	CAD	0.47	0.29
rs10064156	5:102413873	RP11-231G15.1	C	0.19	T2D	0.01	0.35
rs11957829	5:121515195	CTC-441N14.4	G	0.08	stroke	0.64	0.43
rs13359291	5:122476457	PRDM6	G	0.39	BP	0.07	0.71
rs10051787	5:122732236	CEP120	T	0.41	WC	0.28	0.29
rs7701094	5:124317011	LMNB1	C	0.48	BMI	0.89	0.35
rs273909	5:131667353	SLC22A4	G	0.03	CAD	0.94	0.94
rs246600	5:142516897	ARHGAP26	T	0.12	CAD	0.95	0.29
rs9687065	5:148391140	SH3TC2	G	0.24	BP	0.97	0.31
rs6882076	5:156390297	TIMD4	T	0.26	TG;LDL-C;TC	0.94	0.81

rs351855	5:176520243	FGFR4	A	0.42	WHR	0.4	0.75
rs634501	5:180218668	MGAT1	A	0.48	HDL-C	0.68	0.42
rs1173766	5:32804528	CTD-2197M16.1	T	0.35	BP	0.17	0.58
rs702634	5:53271420	ARL15	G	0.12	T2D	0.52	0.47
rs459193	5:55806751	C5orf67	A	0.47	T2D	0.15	0.38
rs3936511	5:55860781	C5orf67	G	0.11	CAD	0.39	0.3
rs6871667	5:74604742	JMY	G	0.45	TC;LDL-C	0.11	0.03
rs191835914	5:74646765	HMGCR	C	0.02	LDL-C	0.98	0.32
rs3846663	5:74655726	HMGCR	C	0.48	TC;LDL-C	0.55	0.28
rs888789	5:74988369	POC5	A	0.46	BMI	0.84	0.37
rs6878122	5:76427311		G	0.05	T2D	0.64	0.47
rs1508798	5:9556694	RP11-260E18.1	C	0.1	CAD	0.86	0.83
rs261967	5:95850250	CTD-2337A12.1	C	0.43	BMI	0.79	0.09
rs9390698	6:101296389	ASCC3	A	0.29	LDL-C;TC	0.73	0.82
rs884366	6:109574095	CCDC162P	A	0.38	HDL-C	0.81	0.57
rs13216675	6:122452329	TRMT11	C	0.4	AF	0.25	0.62
rs1591805	6:126717064	RP11-394G3.2	A	0.02	CAD	0.83	0.63
rs13209747	6:127115454	LOC105377992 - LOC105377991	T	0.48	BP	0.001	0.17
rs9349379	6:12903957	PHACTR1	A	0.28	CAD	0.04	0.85
rs12204590	6:1337393	RP11-157J24.2	A	0.01	stroke	0.04	0.73
rs12202017	6:134173151	TARID	G	0.43	CAD	0.45	0.22
rs12524865	6:134196674	TARID	A	0.42	CAD	0.61	0.25
rs78169666	6:1349830	RP11-157J24.2	C	0	stroke	0.4	0.33
rs9376090	6:135411228	HBS1L	C	0.3	TC	0.15	0.05
rs17080091	6:150997401	PLEKHG1	T	0.06	CAD	0.86	0.96
rs17080102	6:151004770	PLEKHG1	C	0.06	BP	0.9	0.98
rs368123	6:160719593		G	0.45	WC	0.22	0.99
rs3120140	6:160738831		A	0.08	CAD	0.3	0.91
rs10455782	6:160839350	SLC22A3	T	0.24	CAD	0.96	0.08
rs376563	6:160851766	SLC22A3	T	0.39	CAD	0.44	0.59
rs12214416	6:160910517	LPAL2	A	0.02	CAD	0.14	0.4
rs7770628	6:161018174	LPA	C	0.12	TC;LDL-C	0.39	0.26
rs9501744	6:1617143	FOXC1	T	0	CAD	0.31	0.08
rs6909752	6:22612629	ZFP57	A	0.2	CAD	0.33	0.73
rs1799945	6:26091179	HFE	G	0.04	BP	0.06	0.13
rs130071	6:31116210	CCHCR1	A	0.09	TG	0.27	0.01
rs3130501	6:31136453	POU5F1	A	0.29	T2D	0.95	0.46
rs9357121	6:31240479	HLA-B	G	0.17	TC;LDL-C	0.01	0.02
rs9266359	6:31332739	DHFRP2	T	0.41	BP	0.18	0.76
rs2021783	6:32044851	TNXB	T	0.22	BP	0.03	0.02

rs9268402	6:32341353	C6orf10	A	0.4	CAD	0.17	0.18
rs3129853	6:32398648	TBC1D22B	A	0.16	TG	0.06	0.01
rs4713766	6:34244245	NUDT3	A	0.09	BMI	0.87	0.11
rs17609940	6:35034800	ANKS1A	C	0.02	CAD	0.65	0.56
rs1321309	6:36638636	LAP3P2	A	0.31	CAD	0.87	0.73
rs9470794	6:38106844	ZFAND3	C	0.31	T2D	0.5	0.72
rs56336142	6:39134099	ACTG1P9	C	0.16	CAD	0.88	0.86
rs1535500	6:39284050	KCNK16	T	0.46	T2D	0.15	0.54
rs16896398	6:43262704	SLC22A7	T	0.27	stroke	0.66	0.84
rs6905288	6:43758873	VEGFA	G	0.27	HDL-C;CAD;TG	0.03	0.01
rs556621	6:44594159	ACTG1P9	T	0.49	stroke	0.05	0.25
rs9473924	6:50834157	RP11-228O6.2	T	0.28	BMI	0.22	0.17
rs9367716	6:57160572	RNU7-66P	G	0.36	CAD	0.9	0.88
rs1334576	6:7211818	RREB1	G	0.46	WHR	0.09	0.81
rs9505118	6:7290437	SSR1	G	0.43	T2D	0.15	0.34
rs4613862	6:82612271	RP11-379B8.1	C	0.17	CAD	0.56	0.21
rs17477177	7:106411858	CTB-111H14.1	C	0.13	BP	0.51	0.14
rs10953541	7:107244545	BCAP29	T	0.16	CAD	0.54	0.28
rs3807989	7:116186241	CAV1	A	0.29	AF	0.25	0.8
rs975722	7:117332914	CFTR	G	0.36	CAD	0.93	0.27
rs11509880	7:12261911	TMEM106B	G	0.35	CAD	0.01	0.08
rs806215	7:127237312	FSCN3	T	0.36	T2D	0.97	0.06
rs4731420	7:127863295	MIR129-1	C	0.11	T2D	0.54	0.91
rs11556924	7:129663496	ZC3HC1	T	0.05	CAD	0.32	0.85
rs7810507	7:130437476	KLF14	A	0.45	WHR	0.31	0.66
rs13233731	7:130437689	KLF14	A	0.28	T2D	0.28	0.23
rs10237377	7:139757136	PARP12	G	0.44	CAD	0.95	0.02
rs6960043	7:15052860	GTF3AP5	C	0.43	T2D	0.85	0.58
rs3918226	7:150690176	NOS3	T	0	CAD	0.69	0.51
rs4142995	7:17919258	SNX13	G	0.44	HDL-C	0.06	0.36
rs2107595	7:19049388	HDAC9	A	0.33	CAD;stroke	0.8	0.79
rs10267593	7:1937261	MAD1L1	A	0.1	CAD	0.03	0.21
rs12535846	7:25861072	AC003090.1	A	0.4	WHR	0.19	0.08
rs4719841	7:25997536	MIR148A	A	0.4	TG	0.13	0.56
rs4722766	7:28235808	JAZF1-AS1	C	0.5	T2D	0.31	0.66
rs4302748	7:36191699	EEPD1	A	0.09	LDL-C	0.76	0.75
rs10278336	7:44245363	YKT6	G	0.35	T2D	0.88	0.23
rs10260816	7:46010100	LOC102723446	C	0.23	BP	0.65	0.55
rs4917014	7:50305863	AC020743.3	G	0.31	HDL-C	0.03	0.23
rs702485	7:6449272		A	0.14	HDL-C	0.03	0.39
rs4724806	7:6487131	DAGLB	G	0.25	CAD	0.2	0.2
rs35332062	7:73012042	MLXIPL	A	0.12	TG	0.47	0.63
rs42039	7:92244422	CDK6	T	0.02	stroke	0.93	0.86

rs10093110	8:106565414	ZFPM2	A	0.41	CAD	0.64	0.68
rs2245019	8:116622906	TRPS1	C	0.14	HDL-C	0.47	0.26
rs13266634	8:118184783	SLC30A8	T	0.42	T2D	0.24	0.35
rs2954029	8:126490972	RP11-136O12.2	A	0.42	CAD	0.08	0.3
rs11136341	8:145043543	PLEC	G	0.1	TC;LDL-C	0.78	0.92
rs1495741	8:18272881	NAT2	A	0.44	TC;TG	0.94	0.11
rs6997340	8:18286997	NAT2	C	0.47	CAD	0.92	0.1
rs10096633	8:19830921	LPL	T	0.09	HDL-C;TG	0.66	0.83
rs6984210	8:22033615	BMP1	G	0	CAD	0.83	0.71
rs12549902	8:41509259	NKX6-3	A	0.38	T2D	0.14	0.51
rs13277801	8:59353534	UBXN2B	C	0.19	LDL-C;TC	0.03	0.11
rs2081687	8:59388565	CYP7A1	T	0.2	TG;TC;LDL-C	0.04	0.1
rs12679556	8:72514228	RP11-1102P16.1	T	0.25	WHR	0.38	0.36
rs4735692	8:76615663	AC016194.1	A	0.39	BMI	0.85	0.87
rs896854	8:95960511	NDUFAF6	T	0.32	T2D	0.73	0.16
rs17150703	8:9745798	LINC00599	A	0.15	BMI	0.2	0.02
rs10820405	9:106010237	LINC01492	A	0.26	stroke	0.81	0.78
rs2230808	9:107562804	ABCA1	T	0.37	TC;HDL-C	0.54	0.51
rs2066714	9:107586753	ABCA1	T	0.27	HDL-C;TC	0.62	0.81
rs1883025	9:107664301	ABCA1	T	0.21	HDL-C;TC	0.57	0.52
rs2575876	9:107665739	ABCA1	A	0.21	HDL-C;TC	0.72	0.46
rs3887137	9:107698612	RP11-217B7.2	T	0.36	WHR	0.79	0.53
rs944172	9:110517794	AL162389.1	C	0.23	CAD	0.68	0.38
rs4836831	9:123496570	AHCYP2	C	0.47	WC	0.68	0.24
rs885150	9:124420173	DAB2IP	C	0.29	CAD	0.59	0.46
rs2519093	9:136141870	ABO	T	0.22	CAD	0.003	0.1
rs507666	9:136149399	ABO	A	0.22	TC;LDL-C	0.01	0.13
rs579459	9:136154168	ABO	C	0.21	CAD;TC;LDL-C	0.004	0.12
rs11787792	9:139252148	GPSM1	G	0.04	T2D	0.13	0.08
rs10757274	9:22096055	CDKN2B-AS1	G	0.45	CAD	0.94	0.92
rs7859727	9:22102165	CDKN2B-AS1	C	0.34	stroke	0.55	0.57
rs1333042	9:22103813	CDKN2B-AS1	A	0.34	CAD	0.49	0.62
rs2383208	9:22132076	CDKN2B-AS1	G	0.43	T2D	0.94	0.56
rs1575972	9:22301092	RP11-408N14.1	A	0.03	T2D	0.99	0.8
rs10968576	9:28414339	LINGO2	G	0.21	BMI	0.08	0.11
rs16933812	9:36969205	PAX5	G	0.26	BMI	0.11	0.24
rs11142387	9:72998332	KLF9	C	0.32	BMI	0.43	0.87
rs17791513	9:81905590	CHCHD2P9	G	0.06	T2D	0.32	0.11
rs16927668	9:8369533	PTPRD	C	0.5	T2D	0.93	0.77
rs2796441	9:84308948	TLE1	G	0.39	T2D	0.85	0.17

rs1211166	9:87285992	NTRK2	G	0.21	BMI	0.75	0.83
rs10821415	9:97713459	C9orf3	A	0.25	AF	0.87	0.76
rs7917772	10:104487443	SFXN2	A	0.49	WHR	0.51	0.29
rs11191416	10:104604916	PFN1P11	G	0.36	CAD	0.14	0.52
rs4409766	10:104616663	BORCS7	C	0.29	BP	0.31	0.34
rs12415501	10:105324774	NEURL1	T	0.13	AF	0.25	0.71
rs2295786	10:105616482	SH3PXD2A	A	0.32	stroke	0.77	0.5
rs4918072	10:105693644	STN1	A	0.19	CAD	0.7	0.51
rs1129555	10:113910721	GPAM	A	0.32	LDL-C;TC	0.35	1
rs2297991	10:113913222	GPAM	T	0.32	HDL-C;TC	0.28	0.9
rs7903146	10:114758349	TCF7L2	T	0.04	T2D	0.19	0.26
rs11196288	10:115057443	RNU7-165P	G	0.36	stroke	0.27	0.91
rs2782980	10:115781527	ADRB1	T	0.15	BP	0.1	0.27
rs10886471	10:121149403	GRK5	T	0.21	T2D	0.67	0.37
rs7916879	10:12300790	RN7SL232P	G	0.4	CAD	0.71	0.94
rs11257655	10:12307894	RN7SL232P	C	0.44	T2D	0.59	0.93
rs4752700	10:124237612	HTRA1	G	0.44	CAD	0.69	0.75
rs2487928	10:30323892	KIAA1462	A	0.22	CAD	0.26	0.52
rs1870634	10:44480811	LINC00841	T	0.26	CAD	0.61	0.4
rs1832007	10:5254847	AKR1C4	G	0.1	TG	0.75	0.69
rs7897379	10:65301725	REEP3	C	0.37	TG	0.46	0.57
rs12242953	10:70865342	SRGN	A	0.11	T2D	0.66	0.44
rs7901016	10:74637326	MCU	C	0.27	LDL-C	0.51	0.95
rs10824026	10:75421208	SYNPO2L	G	0.39	AF	0.47	0.27
rs12571751	10:80942631	ZMIZ1	G	0.46	T2D	0.14	0.08
rs17680741	10:82251514	TSPAN14	C	0.03	CAD	0.45	0.58
rs1412444	10:91002927	LIPA	T	0.32	CAD	0.87	0.77
rs7087591	10:94473629	Y_RNA	G	0.24	T2D	0.68	0.46
rs2068888	10:94839642	CYP26A1	G	0.18	TG;HDL-C	0.68	0.5
rs9663362	10:95895177	PLCE1	G	0.32	BP	0.66	0.58
rs633185	11:100593538	ARHGAP42	C	0.49	BP	0.97	0.33
rs7947761	11:100624599	ARHGAP42	G	0.02	CAD	0.21	0.64
rs660599	11:102729757	MMP12	A	0.14	stroke	0.01	0.02
rs2128739	11:103673277	RP11-563P16.1	C	0.38	CAD	0.73	0.33
rs180327	11:116623659	BUD13	C	0.35	HDL-C;TG	0.31	0.62
rs964184	11:116648917	ZPR1	G	0.22	CAD	0.22	0.83
rs2075291	11:116661392	APOA5	A	0.06	TG;HDL-C	0.26	0.56
rs651821	11:116662579	APOA5	C	0.28	TC;TG;HDL-C	0.24	0.85
rs12718465	11:116707736	APOA1	T	0.03	HDL-C	0.99	0.75
rs17122278	11:118449370	ARCN1	G	0.47	TC	0.79	0.5
rs17135399	11:126218541	DCPS	G	0.1	LDL-C	0.38	0.53
rs11222084	11:130273230	ADAMTS8	T	0.02	BP	0.04	0.72
rs3993105	11:13303071	ARNTL	C	0.45	CAD	0.01	0.46
rs4757391	11:16302939	SOX6	C	0.28	BP	0.08	0.64

rs2334499	11:1696849	FAM99B	C	0.17	T2D	0.29	0.2
rs5215	11:17408630	KCNJ11	C	0.4	T2D	0.42	0.93
rs7107784	11:2215089	MIR4686	G	0.1	T2D	0.93	0.95
rs117601636	11:2642037	KCNQ1	G	0.09	T2D	0.75	0.9
rs11030104	11:27684517	BDNF	G	0.48	BMI	0.01	0.15
rs2237892	11:2839751	KCNQ1	T	0.32	BMI	0.92	0.52
rs2237896	11:2858440	KCNQ1	A	0.36	T2D	0.66	0.93
rs7116641	11:43696917	RP11-472I20.4	G	0.19	CAD	0.59	0.07
rs326214	11:47298360	MADD	A	0.34	HDL-C	0.1	0.63
rs11604680	11:47457539	RAPSN	G	0.31	BMI	0.32	0.71
rs11601507	11:5701074	TRIM5	A	0.11	CAD	0.47	0.81
rs751984	11:61278246	LRRC10B	C	0.37	BP	0.45	0.23
rs174546	11:61569830	FADS1	T	0.4	HDL-C;TG;TC	0.14	0.95
rs174547	11:61570783	FADS1	C	0.4	HDL-C;TC;TG	0.15	0.92
rs12801636	11:65391317	PCNX3	A	0.44	CAD;HDL-C	0.05	0.1
rs1552224	11:72433098	ARAP1	C	0.09	T2D	0.76	0.95
rs590121	11:75274150	SERPINH1	T	0.19	CAD	0.22	0.81
rs499974	11:75455021	RN7SL786P	A	0.24	HDL-C	0.36	0.11
rs10160804	11:8600240	STK33	A	0.46	BMI	0.23	0.58
rs10830963	11:92708710	MTNR1B	G	0.42	T2D	0.32	0.64
rs93138	11:9759713	SWAP70	G	0.43	CAD	0.78	0.77
rs173396	11:9759918	SWAP70	A	0.43	CAD	0.71	0.55
rs7965082	12:100800193	SLC17A8	T	0.41	LDL-C;TC	0.21	0.66
rs2075260	12:109696838	ACACB	G	0.27	TG	0.31	0.96
rs9593	12:109994870	MMAB	T	0.3	HDL-C	0.43	0.17
rs7134594	12:110000193	MMAB	T	0.3	HDL-C	0.32	0.25
rs12229654	12:111414461	LINC01405	G	0.17	BMI	0.94	0.14
rs6490029	12:111698457	CUX2	G	0.28	AF	0.11	0.06
rs3184504	12:111884608	SH2B3	T	0	stroke;CAD	0.1	0.2
rs671	12:112241766	ALDH2	A	0.19	HDL-C	0.93	0.31
rs11066280	12:112817783	HECTD4	A	0.22	CAD	0.44	0.09
rs10507248	12:114797093	TBX5	T	0.41	AF	0.47	0.68
rs35444	12:115552437	RP11-25E2.1	G	0.23	BP;stroke	0.59	0.31
rs11067763	12:116198341	RP11-110L15.1	G	0.38	BP	0.8	0.38
rs11830157	12:118265441	KSR2	G	0.26	CAD	0.26	0.3
rs1169288	12:121416650	HNF1A	C	0.42	LDL-C;TC	0.64	0.58
rs55783344	12:121432299	HNF1A	T	0.12	T2D	0.3	0.21
rs2258287	12:121454313		C	0.25	CAD	0.57	0.73
rs3213545	12:121471337	OASL	A	0.46	CAD	0.85	0.38
rs4275659	12:123447928	ABCB9	T	0.35	T2D	0.29	0.89
rs10773003	12:123775127	SBNO1	A	0.29	HDL-C	0.39	0.61
rs838880	12:125261593	SCARB1	T	0.5	HDL-C	0.16	0.94

rs11057830	12:125307053	SCARB1	A	0.12	CAD	0.81	0.82
rs12581963	12:125317125	SCARB1	A	0.16	CAD	0.73	0.61
rs7980458	12:20162571	RP11-405A12.2	G	0.45	BP	0.95	0.17
rs3861086	12:20218869	RP11-664H17.1	T	0.39	CAD	0.52	0.6
rs7304841	12:20577593	PDE3A	C	0.39	stroke	0.42	0.72
rs1027087	12:26470850	RP11-283G6.4	T	0.31	WHR	0.1	0.49
rs10842992	12:27963839	RN7SKP15	C	0.35	T2D	0.23	0.01
rs80234489	12:31441179	FAM60A	C	0.16	T2D	0.15	0.41
rs4766228	12:4363420	CCND2-AS1	A	0.5	T2D	0.86	0.11
rs897057	12:50266279	FAIM2	T	0.25	BMI	0.11	0.07
rs7306523	12:53393964	EIF4B	G	0.32	LDL-C;TC	0.15	0.53
rs3809128	12:56709919	CNPY2	T	0.21	WC	0.95	0.9
rs2261181	12:66212318	RPSAP52	T	0.12	T2D	0.57	0.25
rs7955901	12:71433293	CTD-2021H9.2	T	0.33	T2D	0.7	0.23
rs11838267	12:7175872	C1S	C	0.1	CAD	0.19	0.69
rs4883263	12:7649484	CD163	T	0.31	HDL-C	0.05	0.6
rs17249754	12:90060586	ATP2B1	A	0.35	BP	0.82	0.53
rs7136259	12:90081188	ATP2B1	T	0.38	CAD	0.88	0.44
rs4883201	12:9082581	PHC1	G	0.32	TC	0.003	0.29
rs7306455	12:95355541	NDUFA12	A	0.13	CAD	0.75	0.26
rs4923678	12:95368163	NDUFA12	G	0.15	CAD	0.71	0.41
rs34008534	12:95381088	NDUFA12	G	0.15	CAD	0.86	0.34
rs11838776	13:111040681	COL4A2	A	0.09	CAD	0.81	0.61
rs1317507	13:113631780		A	0.23	CAD	0.61	0.41
rs9552911	13:23864657	SGCG	A	0.22	T2D	0.41	0.54
rs9512699	13:28029896	MTIF3	G	0.21	BMI	0.12	0.36
rs9319428	13:28973621	FLT1	A	0.42	CAD	0.01	0.02
rs9534262	13:32936646	BRCA2	T	0.47	LDL-C	0.14	0.47
rs9591012	13:33058333	N4BP2L2	A	0.17	CAD	0.36	0.01
rs1467605	13:47211861	LRCH1	A	0.1	stroke	0.68	0.85
rs9568867	13:54107352	RP11-384G23.1	A	0.25	BMI	0.72	0.67
rs1359790	13:80717156	RP11-470M1.2	A	0.28	T2D	0.35	0.66
rs7989336	13:97017548	HS6ST3	A	0.5	BMI	0.45	0.57
rs10139550	14:100145710	HHIPL1	G	0.31	CAD	0.69	0.72
rs11847697	14:30515112	PRKD1	T	0	BMI	NA	
rs2415317	14:36609678	LINC00609	A	0.43	stroke	0.04	0.14
rs1982963	14:52509101	NID2	G	0.18	WHR	0.08	0.58
rs2145598	14:58794001	ARID4A	A	0.46	CAD	0.32	0.68
rs1152591	14:64680848	SYNE2	A	0.29	AF	0.13	0.26
rs11624704	14:78786077	NRXN3	C	0.06	BMI	0.07	0.32
rs7403531	15:38822905		T	0.36	T2D	0.18	0.6

rs67839313	15:40619724	C15orf52	C	0.18	T2D	0.24	0.3
rs4471613	15:58551694	intergenic (near ALDH1A2, AQP9, LIPC)	A	0.06	stroke	0.13	0.37
rs2043085	15:58680954	ALDH1A2	T	0.47	HDL-C;TG;TC	0.51	0.25
rs1532085	15:58683366		A	0.46	TC;TG;HDL-C	0.58	0.39
rs1077834	15:58723479	LIPC	C	0.39	HDL-C;TC	0.79	0.59
rs1800588	15:58723675	LIPC	T	0.38	TG;TC;HDL-C	0.99	0.78
rs1436953	15:62414014	NPM1P47	T	0.36	T2D	0.03	0.32
rs6494488	15:65024204	RBPMS2	G	0.07	CAD	0.19	0.87
rs56062135	15:67455630	SMAD3	T	0.02	CAD	0.91	0.11
rs4776970	15:68080886	MAP2K5	A	0.23	BMI	0.53	0.12
rs7164883	15:73652174	HCN4	G	0.1	AF	0.92	0.02
rs1378942	15:75077367	CSK	A	0.16	BP	0.14	0.95
rs7178572	15:77747190	HMG20A	G	0.36	T2D	0.16	0.67
rs12438008	15:79084680	ADAMTS7	A	0.38	CAD	0.34	0.54
rs4468572	15:79124475	MORF4L1	T	0.47	CAD	0.27	0.59
rs11634397	15:80432222	ZFAND6	G	0.09	T2D	0.82	0.25
rs8030379	15:84590910	ADAMTSL3	G	0.23	WC	0.18	0.26
rs8042271	15:89574218	RP11-326A19.2	A	0.34	CAD	0.2	0.43
rs2028299	15:90374257	AP3S2	C	0.2	T2D	0.43	0.72
rs4932370	15:91404705	RN7SL363P	A	0.09	stroke	0.56	0.98
rs17514846	15:91416550	FURIN	A	0.15	CAD	0.45	0.78
rs1029420	15:91441086	MAN2A2	C	0.1	BP	0.75	0.88
rs79548680	15:91505779	RCCD1	G	0.5	T2D	0.51	0.13
rs17581137	15:96146414	RP11-61O11.1	C	0.11	CAD	0.5	0.34
rs12597579	16:20257867	GP2	T	0.27	BMI	0.33	0.81
rs4788102	16:28873398	SH2B1	A	0.14	BMI	0.99	0.11
rs2531995	16:4013467	ADCY9	T	0.33	BMI	0.047	<0.001
rs1421085	16:53800954	FTO	C	0.12	T2D	0.69	0.69
rs1558902	16:53803574	FTO	A	0.12	BMI	0.72	0.66
rs247616	16:56989590	AC012181.1	T	0.16	HDL-C;TC	0.54	0.96
rs7499892	16:57006590	CETP	T	0.17	TG;HDL-C;LDL-C	0.9	0.39
rs2303790	16:57017292	CETP	G	0.02	HDL-C	0.37	0.99
rs2292318	16:67985706	SLC12A4	T	0.12	HDL-C	0.7	0.23
rs3785100	16:67997920	SLC12A4	C	0.12	HDL-C	0.72	0.26
rs17358402	16:71967927	PKD1L3	T	0.06	TC;LDL-C	0.78	0.99
rs7185272	16:72013797	PKD1L3	G	0.26	LDL-C;TC	0.5	0.75
rs12927205	16:72025077	PKD1L3	G	0.26	LDL-C;TC	0.55	0.96
rs2000999	16:72108093	HPR	A	0.26	TC;LDL-C	0.7	0.14

rs7193343	16:73029160	ZFHX3	C	0.34	stroke	0.18	0.89
rs2106261	16:73051620	ZFHX3	T	0.33	AF	0.63	0.11
rs12932445	16:73069888	ZFHX3	C	0.36	stroke	0.77	0.29
rs7202877	16:75247245	CTRB1	G	0.19	T2D	0.98	0.56
rs7206541	16:75436713	CFDP1	A	0.48	CAD	0.99	0.4
rs1424233	16:79682751	AC009159.1	C	0.33	BMI	0.06	0.06
rs2925979	16:81534790	CMIP	T	0.41	HDL-C	0.58	0.3
rs7199941	16:81906423	PLCG2	A	0.5	CAD	0.69	0.92
rs7500448	16:83045790	CDH13	G	0.24	CAD	0.7	0.4
rs12445022	16:87575332	RP11-482M8.1	A	0.09	stroke	0.02	0.05
rs12936587	17:17543722	RP11-524F11.3	A	0.11	CAD	0.98	0.65
rs216172	17:2126504	SMG6	C	0.26	CAD	0.27	0.41
rs391300	17:2216258	SRR	T	0.29	T2D	0.96	0.85
rs13723	17:27941886	CORO6	G	0.3	CAD	0.28	0.68
rs76954792	17:30033514	RP11-805L22.1	T	0.07	CAD	0.05	0.12
rs11651052	17:36102381	HNF1B	A	0.27	T2D	0.6	0.3
rs7208487	17:37543449	FBXL20	G	0.27	HDL-C	0.13	0.89
rs11869286	17:37813856	STARD3	C	0.44	HDL-C	0.05	0.63
rs2074158	17:40257163	DHX58	C	0.12	CAD	0.81	0.77
rs12946454	17:43208121	PLCD3	T	0.21	BP	0.02	0.25
rs17608766	17:45013271	GOSR2	C	0	CAD	0.82	0.49
rs9299	17:46669430	HOXB5	C	0.47	BMI	0.09	0.03
rs7405452	17:46674670	HOXB6	T	0.17	BP	0.47	0.97
rs46522	17:46988597	UBE2Z	C	0.28	CAD	0.99	0.81
rs7213603	17:59003189	BCAS3	C	0.13	CAD	0.88	0.65
rs7225581	17:59017813	BCAS3	A	0.13	CAD	0.84	0.68
rs2240736	17:59485393	TBX2	T	0.44	BP	0.8	0.27
rs1867624	17:62387091	RPL31P57	C	0.21	CAD	0.63	0.81
rs9892152	17:62401965	RPL31P57	T	0.33	CAD	0.36	0.81
rs4148008	17:66875294	ABCA8	G	0.43	HDL-C	0.49	0.97
rs12453914	17:67138878	ABCA6	A	0.42	LDL-C;TC	0.18	0.45
rs11077501	17:68526061	RP11-1058G23.1	C	0.29	WHR	0.11	0.58
rs13342232	17:6945940	SLC16A11	G	0.12	T2D	0.61	0.52
rs4129767	17:76403984	PGS1	A	0.33	HDL-C	0.67	0.13
rs7503807	17:78591111	RPTOR	C	0.3	BMI	0.72	0.65
rs1805081	18:21140432	NPC1	C	0.23	BMI	0.81	0.56
rs2000813	18:47093864	LIPG	T	0.29	HDL-C	0.84	0.73
rs12970066	18:47107152	LIPG	G	0.28	HDL-C	0.68	0.59
rs4939883	18:47167214	SMUG1P1	T	0.18	HDL-C;TC	0.46	0.9
rs2156552	18:47181668	SMUG1P1	A	0.18	HDL-C	0.86	0.92
rs11660468	18:47209143	SMUG1P1	T	0.22	CAD	0.88	0.58
rs35337492	18:47210824	SMUG1P1	A	0.22	CAD	0.86	0.46
rs663129	18:57838401	RNU4-17P	A	0.21	CAD	0.94	0.1

rs8090011	18:7068462		C	0.3	T2D	0.44	0.17
rs2229383	19:10794630	ILF3-SLC44A2	G	0.4	stroke	0.77	0.75
rs7258189	19:11169947	SMARCA4	C	0.12	stroke	0.86	0.98
rs56289821	19:11188247	LDLR	A	0.01	CAD	0.9	0.74
rs200990725	19:11217315	LDLR	T	0	TC;LDL-C	0.37	0.93
rs7258950	19:11250139	SPC24	A	0.22	LDL-C;TC	0.94	0.76
rs11557092	19:11257018	SPC24	T	0.26	TC;LDL-C	0.07	0.52
rs737337	19:11347493	DOCK6	C	0.28	LDL-C;HDL-C;TC	0.83	0.74
rs73015714	19:17855763	FCHO1	G	0.03	CAD	0.47	0.08
rs58542926	19:19379549	TM6SF2	T	0.07	TC;TG;LDL-C	0.57	0.7
rs10401969	19:19407718	SUGP1	C	0.1	T2D	0.31	0.68
rs740406	19:2232221		G	0.3	BP	0.56	0.23
rs16967013	19:32859514	ZNF507	G	0.4	CAD	0.67	0.53
rs29941	19:34309532	KCTD15	G	0.23	BMI	0.89	0.26
rs7258445	19:41855515	TGFB1	A	0.39	CAD	0.05	0.55
rs769449	19:45410002	APOE	A	0.09	TG;HDL-C;LDL-C;TC	0.54	0.84
rs72654473	19:45414399	APOC1	A	0.08	TC;TG	0.51	0.19
rs439401	19:45414451	APOC1	C	0.41	HDL-C;TC;TG	0.92	0.65
rs4420638	19:45422946	APOC1	G	0.12	CAD	0.64	0.92
rs8108269	19:46158513	RN7SL836P	T	0.5	T2D	0.69	0.56
rs2302593	19:46196634	GIPR	G	0.42	CAD	0.09	0.16
rs3810291	19:47569003	ZC3H4	A	0.3	BMI	0.84	0.21
rs17695224	19:52324216	FPR3	A	0.19	HDL-C	0.38	0.9
rs1887320	20:10965998	RP11-103J8.1	G	0.47	BP	0.73	0.16
rs2328223	20:17845921	RIN2	C	0.2	LDL-C	0.55	0.79
rs867186	20:33764554	PROCR	G	0.06	CAD	0.09	0.06
rs4911495	20:33971978	UQCC1	C	0.16	WHR	0.57	0.79
rs6065311	20:39724338	TOP1	T	0.18	LDL-C	0.89	0.88
rs6093446	20:39780932	PLCG1	A	0.3	CAD	0.96	0.05
rs4812829	20:42989267	HNF4A	A	0.44	T2D	0.65	0.33
rs3827066	20:44586023	ZNF335	T	0.06	CAD	0.1	0.02
rs16990971	20:44601293	ZNF335	G	0.06	TG	0.7	0.29
rs13041126	20:51092996	LINC01524	C	0.3	BMI	0.17	0.61
rs2057291	20:57472043	GNAS	A	0.27	CAD;WC	0.57	0.78
rs6038557	20:6594349	RP5-971N18.3	G	0.19	WC	0.46	0.37
rs181359	22:21928641	UBE2L3	A	0.47	HDL-C	0.21	0.48
rs181360	22:21928916	UBE2L3	G	0.47	HDL-C	0.22	0.44
rs16999793	22:24656875	POM121L9P	C	0.1	CAD	0.41	0.32
rs4821382	22:35638115	SCUBE1	G	0.38	WHR	0.57	0.19
rs5996074	22:42236337	SREBF2	G	0.46	BMI	0.38	0.28

rs738409	22:44324727	PNPLA3	G	0.45	TG	0.36	0.28
rs1800234	22:46615880	PPARA	C	0.05	TG	0.02	0.1

Significant interactions were colored red at *P*<0.001. No statistically significant multiplicative and additive interactions between each single SNP included in the PRS and fruit intake were observed after correction for multiple testing (Bonferroni-adjusted significance threshold of *P*<0.05/534).

Abbreviations: AF, atrial fibrillation; AP, attributable proportion due to the interaction; BMI, body mass index; BP, blood pressure; CAD, coronary artery disease; CI, confidence interval; DBP, diastolic blood pressure; HDL-C, high-density lipoprotein cholesterol; LDL-C, low-density lipoprotein cholesterol; MAF, minor allele frequency; MAP, mean arterial pressure; PP, pulse pressure; RERI, relative excess risk due to interaction; SBP, systolic blood pressure; SNP, single nucleotide polymorphism; T2D, type 2 diabetes; TC, total cholesterol; TG, triglycerides; WC, waist circumference; WHR, waist hip ratio.

Table S2. Baseline characteristics by the genetic risk score and the daily fruit intake

Characteristic	Genetic risk score			*P* value	Fruit intake			*P* value
	Low (n=6974)	Intermediate (n=20,923)	High (n=6974)		<200 g/week (n=11,928)	200 g/week–100 g/day (n=12,867)	>100 g/day (n=10,076)	
Age, year	55±10	55±10	55±10	<0.001	56±10	55±10	55±10	<0.001
Female, n (%)	4047 (58)	12,018 (57)	4063 (58)	0.41	6888 (58)	7382 (57)	5858 (58)	0.51
Northern, n (%)	2393 (34)	10,243 (49)	4190 (60)	<0.001	3391 (33)	6909 (54)	5926 (59)	<0.001
Urban residence, n (%)	1266 (18)	3350 (16)	997 (14)	<0.001	992 (8.3)	1817 (14)	2804 (28)	<0.001
High-school or above, n (%)	3227 (46)	9771 (47)	3325 (48)	0.23	4308 (36)	6205 (48)	5810 (58)	<0.001
Current smoker, n (%)	1637 (24)	4968 (24)	1588 (23)	0.24	3081 (26)	2947 (23)	2165 (22)	<0.001
Alcohol drinker, n (%)	1310 (19)	4996 (24)	1786 (26)	<0.001	2906 (24)	2860 (22)	2326 (23)	<0.001
Family history of stroke, n (%)	664 (9.5)	2056 (9.8)	771 (11)	0.004	1118 (9.4)	1237 (9.6)	1136 (11)	<0.001
Ideal physical activity, n (%)	4305 (62)	13,180 (63)	4421 (63)	0.09	7886 (66)	8120 (63)	5900 (59)	<0.001
Ideal diet score, n (%)	3763 (54)	10,800 (52)	3542 (51)	<0.001	5421 (45)	6719 (52)	5965 (59)	<0.001
Body mass index, kg/m^2	24±4	24±4	24±4	<0.001	24±4	24±4	24±4	<0.001
Waist circumstance, cm	81±10	82±10	83±10	<0.001	81±10	82±10	83±10	<0.001
Hypertension, n (%)	2273 (33)	7982 (38)	3125 (45)	<0.001	4371 (37)	5005 (39)	4004 (40)	<0.001
Diabetes mellitus, n (%)	512 (7.5)	1724 (8.4)	689 (10)	<0.001	1059 (9.1)	1044 (8.3)	822 (8.4)	0.06
Dyslipidemia, n (%)	1988 (29)	6616 (32)	2468 (36)	<0.001	3654 (31)	4128 (33)	3290 (34)	0.003

Mean ± standard deviation for continuous variables and numbers (percentages) for dichotomous variables. Ideal physical activity level was defined as at least 150 minutes/week of moderate physical activity or 75 minutes/week of vigorous physical activity or an equivalent combination. The ideal diet was defined as healthy diet score ≥2 components: red meat <75 g/day, legumes ≥125 g/day, fish ≥200 g/week, and tea ≥3 times/week.

Table S3. Association of the stroke risk with the sociodemographic and behavioral factors[*]

**Character**	**HR (95% CI)**	***P* value**
Age	1.069 (1.065–1.074)	<0.001
Sex	0.81 (0.75–0.87)	<0.001
Northern	1.28 (1.18–1.39)	<0.001
Urbanization	0.75 (0.62–0.91)	0.003
High-school or above	0.54 (0.50–0.59)	<0.001
Current smoker	1.17 (1.07–1.29)	<0.001
Alcohol drinker	0.92 (0.84–1.02)	0.11
Family history of stroke	1.50 (1.33–1.70)	<0.001
Ideal physical activity	0.85 (0.78–0.92)	<0.001
Ideal diet score	0.73 (0.68–0.79)	<0.001
Body mass index	1.02 (1.00–1.03)	<0.001
Vegetable intake	1.04 (0.96–1.14)	0.31

[*]Univariate Cox regression model was used.

Table S4. Multivariable-adjusted HRs (95%CIs) for genetic risk, fruit intake and incident stroke

Analysis	Category	No. of events	Total No.	Person-time at risk	Incidence[§]	Base model		Full model	
						HR (95% CI)	*P* trend	HR (95% CI)	*P* trend
Genetic risk[*]	Low	416	6974	68,403	6.6 (5.9–7.3)	Reference	<0.001	Reference	<0.001
	Intermediate	1535	20,923	202,045	7.7 (7.3–8.2)	1.19 (1.06–1.32)		1.15 (1.03–1.29)	
	High	635	6974	66,862	8.6 (8.0–9.3)	1.51 (1.33–1.71)		1.44 (1.27–1.63)	
Fruit intake[#]	<200 g/week	1098	11,928	115,692	8.4 (7.9–9.0)	Reference	<0.001	Reference	<0.001
	200 g/week–100 g/day	952	12,867	125,755	7.8 (7.3–8.3)	0.81 (0.74–0.89)		0.81 (0.74–0.89)	
	>100 g/day	536	10,076	95,864	6.6 (6.1–7.2)	0.68 (0.61–0.76)		0.69 (0.62–0.77)	

[§]Age- and sex-adjusted incidence rates per 1000 person-years of stroke were calculated using Poisson regression.

HRs were derived from the Cox proportional hazards model stratified by cohort.

[*]Base model for genetic risk was adjusted for age (continuous), sex (male, female). Full model was further adjusted for region (North/South China), urbanization (urban, rural), education level (less than high school, high school or above), current smoking status (yes, no), alcohol drinking status (yes, no), physical activity (continuous), body mass index (continuous), diet score (continuous), and vegetables intake (daily intake ≥500 g/day or not).

[#]Base model for fruit intake was adjusted for age (continuous), sex (male, female), region (North/South China), urbanization (urban, rural), and education level (less than high school, high school or above). Full model was further adjusted for current smoking status (yes, no), alcohol drinking status (yes, no), physical activity (continuous), body mass index (continuous), diet score (continuous), and vegetables intake (daily intake ≥500 g/day or not).

Abbreviations: CI, confidence interval; HR, hazard ratio.

Table S5. Sensitivity analysis of the fruit intake associated with incident stroke, according to the genetic risk category

	Using chronological age as the time-scale[*]		Using competing risk model [#]		Adjusting smoking pack year and daily alcohol intake[†]		Further adjusting family history of stroke[‡]		Further adjusting per-capita household income[⁋]	
	**HR (95% CI)**	***P* trend**	**HR (95% CI)**	***P* trend**	**HR (95% CI)**	***P* trend**	**HR (95% CI)**	***P* trend**	**HR (95% CI)**	***P* trend**
**Low genetic risk**										
<200 g/week	Reference	0.048	Reference	0.03	Reference	0.02	Reference	0.04	Reference	0.04
200 g/week–100 g/day	0.84 (0.67–1.06)		0.85 (0.67–1.07)		0.81 (0.64–1.03)		0.81 (0.64–1.02)		0.82 (0.65–1.04)	
>100 g/day	0.74 (0.56–0.98)		0.72 (0.55–0.95)		0.70 (0.52–0.93)		0.72 (0.55–0.96)		0.72 (0.54–0.96)	
**Intermediate genetic risk**										
<200 g/week	Reference	<0.001	Reference	<0.001	Reference	<0.001	Reference	<0.001	Reference	<0.001
200 g/week–100 g/day	0.82 (0.73–0.93)		0.82 (0.72–0.92)		0.81 (0.72–0.92)		0.83 (0.74–0.93)		0.82 (0.73–0.92)	
>100 g/day	0.68 (0.59–0.79)		0.68 (0.59–0.79)		0.66 (0.57–0.77)		0.69 (0.60–0.80)		0.68 (0.58–0.78)	
**High genetic risk**										
<200 g/week	Reference	0.002	Reference	0.005	Reference	0.01	Reference	0.006	Reference	0.005
200 g/week–100 g/day	0.82 (0.68–0.99)		0.82 (0.68–0.99)		0.78 (0.64–0.94)		0.82 (0.68–0.99)		0.82 (0.68–0.99)	
>100 g/day	0.69 (0.55–0.86)		0.71 (0.56–0.88)		0.71 (0.57–0.89)		0.72 (0.58–0.89)		0.71 (0.57–0.88)	

HRs were derived from the Cox proportional hazards model stratified by cohort.
[*]Adjusted for sex, region, urbanization, education level, current smoking status, alcohol drinking status, physical activity, body mass index, diet score, and vegetables intake. [#]Adjusted for age, sex, region, urbanization, education level, current smoking status, alcohol drinking status, physical activity, body mass index, diet score, and vegetables intake. [†] Adjusted for smoking pack year and daily alcohol intake (g ethanol/day) instead of categorical variables. [‡] Adjusted for age, sex, region, urbanization, education level, current smoking status, alcohol drinking status, physical activity, body mass index, diet score, vegetables intake, and family history of stroke. [⁋] Adjusted

for age, sex, region, urbanization, education level, current smoking status, alcohol drinking status, physical activity, body mass index, diet score, vegetables intake, and per-capita household income. Abbreviations: CI, confidence interval; HR, hazard ratio.

Table S6. Risk of incident stroke, according to the fruit intake frequency and genetic risk

	No. events/ Total No.	Incidence*	Full model: HR (95% CI)	Full model: *P trend*	Full model: *P* interaction	Absolute risk over 10 years (95% CI)	Absolute risk reduction over 10 years	*P* trend
**Low genetic risk**				<0.001				
Never or rarely	208 / 2469	7.8 (6.8–9.0)	Reference		*P* multiplicative interaction =0.89	5.2% (4.3–6.1)	0 (Reference)	
Weekly	135 / 2708	6.0 (5.0–7.1)	0.64 (0.51–0.81)			3.4% (2.7–4.0)	1.8%	
Daily	73 / 1797	5.7 (4.5–7.2)	0.57 (0.42–0.76)			2.9% (2.2–3.7)	2.3%	
**Intermediate genetic risk**				<0.001	*P* additive interaction =0.65 RERI=0.07 (95% CI: −0.21–0.34) AP=0.04 (95% CI: −0.10–0.17)			
Never or rarely	770 / 7547	9.4 (8.7–10.2)	Reference			6.0% (5.4–6.5)	0 (Reference)	0.03
Weekly	528 / 7922	7.2 (6.6–7.9)	0.75 (0.67–0.84)			4.5% (4.0–4.9)	1.5%	
Daily	237 / 5454	6.0 (5.3–6.8)	0.56 (0.48–0.66)			3.4% (2.9–3.8)	2.6%	
**High genetic risk**				<0.001				
Never or rarely	308 / 2550	9.9 (8.8–11.1)	Reference			8.3% (7.2–9.4)	0 (Reference)	
Weekly	219 / 2542	8.1 (7.1–9.2)	0.75 (0.63–0.90)			6.3% (5.4–7.2)	2.0%	
Daily	108 / 1882	7.8 (6.4–9.4)	0.53 (0.42–0.67)			4.5% (3.6–5.4)	3.8%	

*Age- and sex-adjusted incidence rates per 1000 person-years of stroke were calculated using Poisson regression.

'Never or rarely' consumers refer to participants whose fruit intake was zero or who eats less than three times per month. 'Weekly' consumers were defined as a person who eats 1–6 times per week. 'Daily' consumers were defined as a person who eats fruit every day.

HRs were derived from the Cox proportional hazards model stratified by cohort and adjusted for age (continuous), sex (male, female), region (North/South China), urbanization (urban, rural), education level (less than high school, high school or above), current smoking status (yes, no), alcohol drinking status (yes, no), physical activity (continuous), body mass index (continuous), diet score (continuous), and vegetables intake (daily intake ≥500 g/day or not). Multiplicative interaction was evaluated using hazard ratios for the product term between the fruit intake frequency (never or rarely vs daily) and PRS (low vs high). To estimate the additive interaction, participants with fruit intake frequency of daily or weekly and the low genetic risk (quintile 1 of PRS) were used as the reference with consideration of practical interpretation. Two indexes were calculated: relative excess risk due to interaction (RERI) and attributable proportion due to the interaction (AP). Abbreviations: CI, confidence interval; HR, hazard ratio; PRS, polygenic risk score.

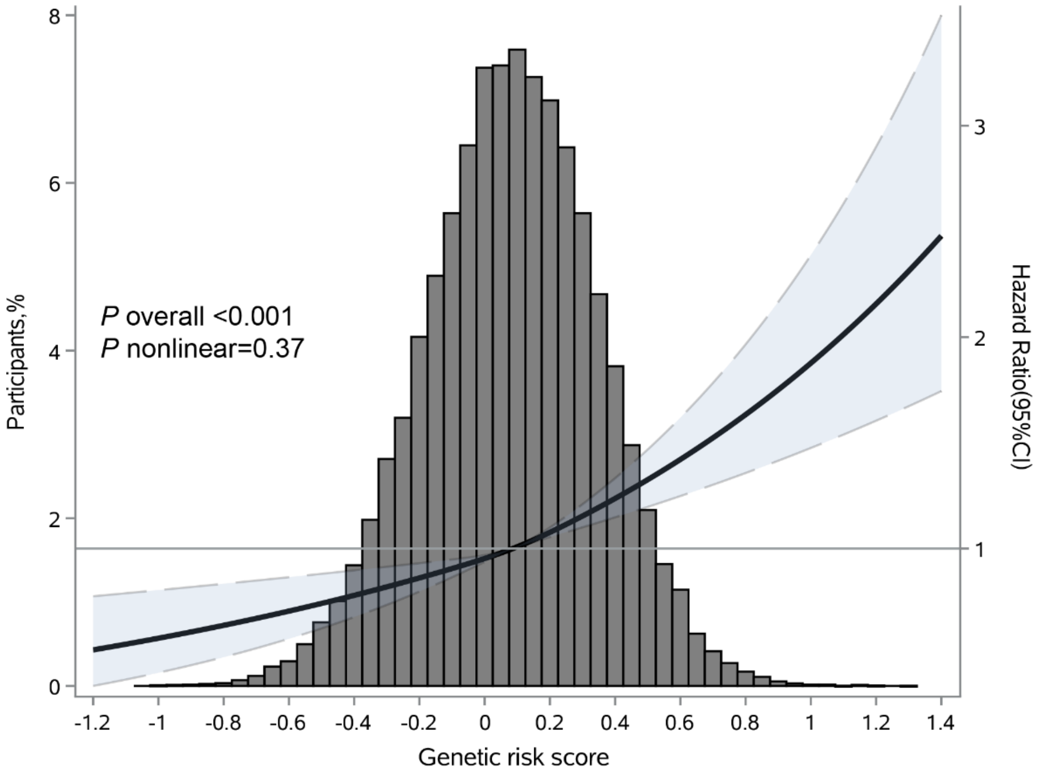

Figure S1. Associations between the genetic risk score and incident stroke. Data were fitted by restricted cubic spline Cox proportional hazards models, with three knots placed at 25th, 50th (reference), and 75th percentiles of genetic risk score as a continuous variable. Solid curves represent point estimates and dashed curves represent 95% CI. Model was adjusted for age (continuous), sex (male, female), and cohort. Abbreviations: CI, confidence interval.

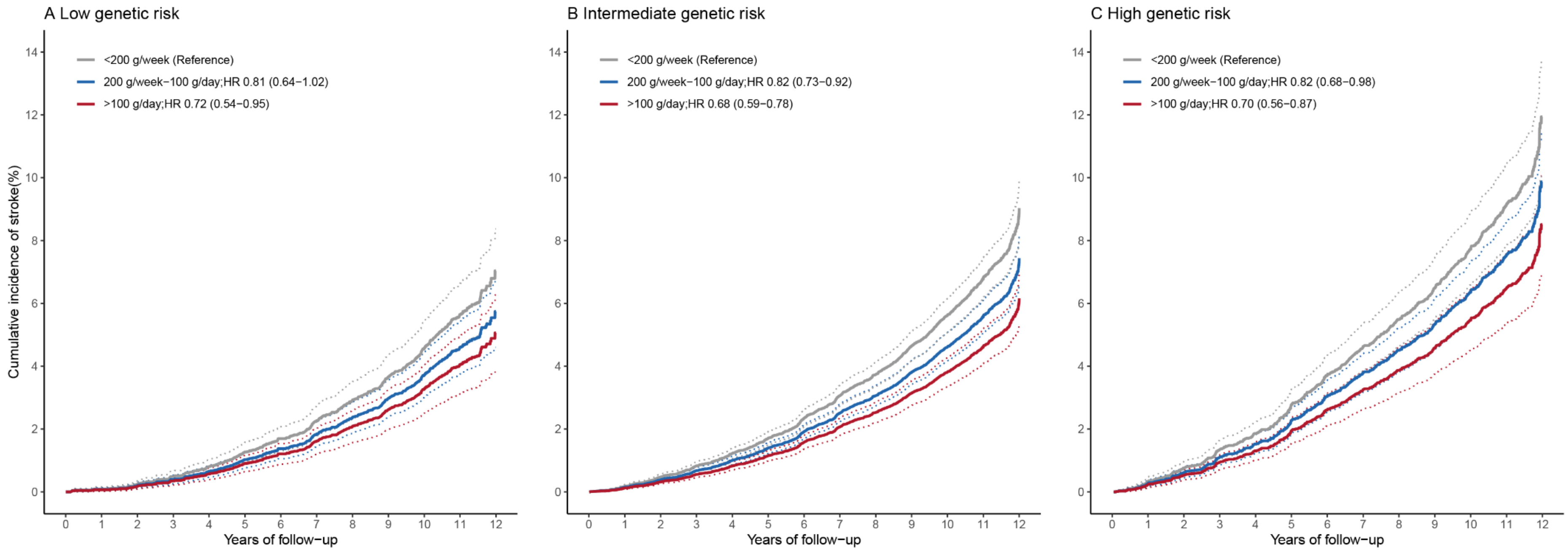

Figure S2. Cumulative incidence of stroke, according to the categories of the genetic risk and fruit intake. Solid lines represented point estimates and dashed lines were 95% confidence intervals. The overall genetic risk for stroke was defined as high (quintile 5 of PRS), intermediate (quintile 2–4 of PRS), and low (quintile 1 of PRS). Models were adjusted for cohort, age, sex, region, urbanization, education level, current smoking status, alcohol drinking status, physical activity, body mass index, diet score, and vegetables intake, which was performed on population averages for each covariate. Abbreviations: HR, hazard ratio; PRS, polygenic risk score.
